# *Ascophyllum nodosum* Extract Improves Olive Performance Under Water Deficit Through the Modulation of Molecular and Physiological Processes

**DOI:** 10.3390/plants13202908

**Published:** 2024-10-17

**Authors:** Maria Celeste Dias, Rui Figueiras, Marta Sousa, Márcia Araújo, José Miguel P. Ferreira de Oliveira, Diana C. G. A. Pinto, Artur M. S. Silva, Conceição Santos

**Affiliations:** 1 Center for Functional Ecology, Associate Laboratory TERRA, Department of Life Sciences, University of Coimbra, Calçada Martim de Freitas, 3000-456 Coimbra, Portugal; rppaf9801@gmail.com (R.F.); marta.mfs.2008@hotmail.com (M.S.); araujo.marciaf@gmail.com (M.A.); 2 LAQV/REQUIMTE, Department of Chemistry, Campus Universitário de Santiago, University of Aveiro, 3810-193 Aveiro, Portugal; diana@ua.pt (D.C.G.A.P.); artur.silva@ua.pt (A.M.S.S.); 3 IB2 Lab, LAQV/REQUIMTE, Department of Biology, Faculty of Sciences, University of Porto, Rua do Campo Alegre, 4169-007 Porto, Portugal; csantos@fc.up.pt; 4 CITAB, University of Trás-os-Montes and Alto Douro, 5001-801 Vila Real, Portugal; 5 LAQV/REQUIMTE, Laboratory of Applied Chemistry, Department of Chemical Sciences, Faculty of Pharmacy, University of Porto, 4169-007 Porto, Portugal; oliveira.miguel@fc.up.pt

**Keywords:** aquaporins, biostimulants, dehydrins, drought, heat shock proteins, photosynthesis

## Abstract

The olive tree is well adapted to the Mediterranean climate, but how orchards based on intensive practices will respond to increasing drought is unknown. This study aimed to determine if the application of a commercial biostimulant improves olive tolerance to drought. Potted plants (cultivars Arbequina and Galega) were pre-treated with an extract of *Ascophyllum nodosum* (four applications, 200 mL of 0.50 g/L extract per plant), and were then well irrigated (100% field capacity) or exposed to water deficit (50% field capacity) for 69 days. Plant height, photosynthesis, water status, pigments, lipophilic compounds, and the expression of stress protective genes (*OeDHN1*—protective proteins’ dehydrin; *OePIP1.1*—aquaporin; and *OeHSP18.3*—heat shock proteins) were analyzed. Water deficit negatively affected olive physiology, but the biostimulant mitigated these damages through the modulation of molecular and physiological processes according to the cultivar and irrigation. *A. nodosum* benefits were more expressive under water deficit, particularly in Galega, promoting height (increase of 15%) and photosynthesis (increase of 34%), modulating the stomatal aperture through the regulation of *OePIP1.1* expression, and keeping *OeDHN1* and *OeHSP18.3* upregulated to strengthen stress protection. In both cultivars, biostimulant promoted carbohydrate accumulation and intrinsic water-use efficiency (iWUE). Under good irrigation, biostimulant increased energy availability and iWUE in Galega. These data highlight the potential of this biostimulant to improve olive performance, providing higher tolerance to overcome climate change scenarios. The use of this biostimulant can improve the establishment of younger olive trees in the field, strengthen the plant’s capacity to withstand field stresses, and lead to higher growth and crop productivity.

## 1. Introduction

Extreme weather events due to climate change are becoming more common and climate projections point to an increase in the frequency and severity of droughts, heatwaves, and floods [1]. The Mediterranean region is particularly vulnerable to the ongoing increased events of drought, which together with water scarcity threaten the agriculture sector, and negatively affect the yield and productivity of several crops [2]. Drought induces several physiological and metabolic changes, and survival under these conditions depends on the development of adaptive and protective strategies [3]. Stomatal control is pivotal to regulate water content but may limit the internal CO_2_ availability and compromise photosynthesis [4,5]. Moreover, an increase in energy-dissipation processes under stress can also prevent photoinhibition damage [6]. Other strategies like adjustments in metabolite biosynthesis and translocation (e.g., amino acids for osmoregulation), and specific stress-responsive proteins, also represent protective responses under drought [6].

The olive tree (*Olea europaea* L.) is one of the main crops of the Mediterranean region. This species has evolved several morphological and physiological adaptations to cope with the harsh climate of this region, such as an efficient ability to regulate transpiration and stomatal aperture, high water uptake capacity, leaf anatomical alterations, and osmotic adjustment [7,8]. Although well adapted to the prevailing climate, several studies demonstrated that stressful events threaten this culture, reducing carbon assimilation, growth, and productivity and changing olive oil quality [2,3]. Several reports described reductions in olive plant water status, stomatal closure, loss of photosynthetic pigments, photoinhibition, and net CO_2_ assimilation impairment in response to water deficit stress [9,10,11,12,13]. Moreover, water deficit can induce oxidative stress and increase investment in defense mechanisms (e.g., antioxidant enzymes and metabolites), leading to considerable expenses for energy and reduction in olive growth and productivity [3,6,14]. Traditional olive orchards rely on rain-fed, dry land culture, but increasing droughts have forced farmers to introduce irrigation. Also, to maintain the world’s increasing demand for olive oil, more productive and profitable orchard practices have been implemented using high-density intensive systems with high levels of irrigation and agrochemicals. However, under the current scenario of extreme climate events, these agricultural systems will be unsustainable, urging the implementation of strategies that enhance plant resilience and improve the efficiency of water management under stress conditions to guarantee production [15,16].

Bio-based products, including biostimulants, can be sustainable alternative agronomic tools to cope with the agricultural challenges imposed by climatic stressors [15,16]. Biostimulants are natural substances or microorganisms that can enhance plant growth and yield, as well as (a)biotic stress tolerance [15,16]. The specific mechanisms behind the beneficial effects of the various types of biostimulants in plants are not well understood; there is evidence that they act as priming agents, activating a complex of molecular and physiological processes that lead to higher performance and tolerance to stresses [15,16]. Priming effects on plants are thought to be triggered by the active compounds present in biostimulants, allowing a more effective response against the various stresses, depending on the type of stress [15,16]. The beneficial effects of biostimulants on plant growth and production under drought conditions have been shown for some important crop/tree species, such as soybean, rice, tomato, maize, grapevine, orange, and lemon trees [16]. However, the effects of biostimulants on olive culture are little known. Chouliaras et al. [17] showed that the application of an extract derived from fronds and strap-like blades of *A. nodosum* in rain-fed olive orchards improved productivity and oil quality, whilst Almadi et al. [18] demonstrated that protein hydrolysates increased photosynthesis in young well-watered potted olive plants, but did not affect fruit and oil yield. Also, biostimulants based on kaolin, glycine betaine, and *Pseudomonas reactans* improved olive performance under drought conditions [19,20].

Biostimulants based on the brown alga *A. nodosum* are amongst the most studied and commercialized extracts [21]. *A. nodosum* is a source of alginate, fucoidan, carrageenan, laminaran, ascophyllan, vitamins, fatty acids, hormones, sterols, minerals, and several phenolic compounds, such as phlorotannins [22]. The promoting effects of *A. nodosum* extracts on photosynthesis, growth, and antioxidant system have been documented in several species, but the mode of action has not yet been completely characterized [15,16]. The composition of *A. nodosum* extracts is complex and depends on the extraction methods, making it difficult to identify the components that play a key role [22]. A commercial biostimulant based on acid extract of *A. nodosum* has been studied in the model species *Arabidopsis thaliana* and data suggested that they act at the stomata level, regulating stomatal conductance by downregulating the expression of genes related to water transport and CO_2_ diffusion in the mesophyll cell (e.g., *PIP1*, and *PIP2*), leading to partial stomatal closure [23]. This effect also helps to maintain adequate plant water content under water deficit and potentially improves the water-use efficiency in some species [24]. Moreover, under stressful conditions, application of this acid extract in *A. thaliana* was shown to protect the photosynthetic apparatus, and promoted carbon assimilation and starch synthesis by regulating the expression of genes related to RuBisCO activation (*RbCS1A* and *RCA*). The acid extract of *A. nodosum* also interfered with the development of protective reactions to dehydration, by upregulating some dehydrins (e.g., *RAB18*) [21] and promoting the uptake of nutrients, leading to higher availability of S and N, and growth of *Brassica napus* [25]. In addition, alkaline and neutral extracts of *A. nodosum* promoted the biosynthesis of pigments, carbohydrates, amino acids, sugar alcohols, and proteins in *Phaseolus vulgaris*, *Spinacia oleracea*, *Capsicum annuum*, *Solanum lycopersicum,* and *A. thaliana* under drought, salt, and cold stress [16,25]. Moreover, the beneficial effects of commercial products based on *A. nodosum* extracts and formulation were also studied in other species under different environmental conditions. In an apple (*Malus domestica*) orchard, Mousavi et al. [26] reported that the application of a commercial alkaline extract of *A. nodosum* promoted tree performance through the enhancement of the photosynthetic pigment contents (chlorophyll and carotenoids), water status, minerals (N, P and Fe), proteins, and antioxidant response (proline, and catalase and peroxidase activities). Also, the yield and fruit quality (fruit weight and diameter) were improved by this biostimulant. Similarly, Ayub et al. [27] tested the application of a commercial alkaline extract of *A. nodosum* with potassium in an apple orchard during two cycles and reported an increase in yield, fruit growth, and quality. In pistachio trees (*Pistacia vera* L.), the acid extract of *A. nodosum* improved the tree antioxidant system (increased the activity of catalase, superoxide dismutase and ascorbate peroxidase in leaves) and fruit nutritional quality [28]. The application of *A. nodosum* extract alone or mixed with calcium led to improved photosynthesis, water status and yield of sweet cherry (*Prunus avium* L.) from an orchard [29]. The use of a commercial formulation containing extracts of seaweed (*A. nodosum* and *Laminaria digitata*) and yeast was tested in *Solanum lycopersicum* plants [30] and also showed beneficial effects in this species. This formulation was effective in mitigating water deficit negative effects on tomato plants growing in pots under greenhouse conditions, decreasing the levels of abscisic acid, malondialdehyde, and proline in leaves.

To maintain the high productivity of the olive orchards under the scenario of climate change and associated water limitations, there is an urgent need to find sustainable strategies to increase plant performance and stress tolerance. Therefore, in this study, we aim to unravel the potential beneficial effects of pre-treating olive plants with a biostimulant based on the seaweed *A. nodosum* (Fitoalgas Green^®^) on olive tolerance to water deficit stress. We hypothesized that the treatment with *A. nodosum* extract before a water deficit episode would improve the functional plasticity by activating protective mechanisms that increase the plant’s physiological capacity to cope with drought stress. To test our hypothesis, olive plants of two cultivars, Galega, usually used in traditional rain-fed orchards, and Arbequina, more frequently used in high-density orchards, were treated with a commercial biostimulant based on *A. nodosum* acid extract and then exposed to different irrigation levels (well-watered and water deficit). The plant height, water status, photosynthesis, pigments, lipophilic metabolites, and the expression of genes related to stress protection, including aquaporins dehydrins, and small heat shock proteins, were analyzed.

## 2. Results

### 2.1. Plant Water Status, Gas Exchange, Chlorophyll a Fluorescence, and Pigments

**Figure 1:**
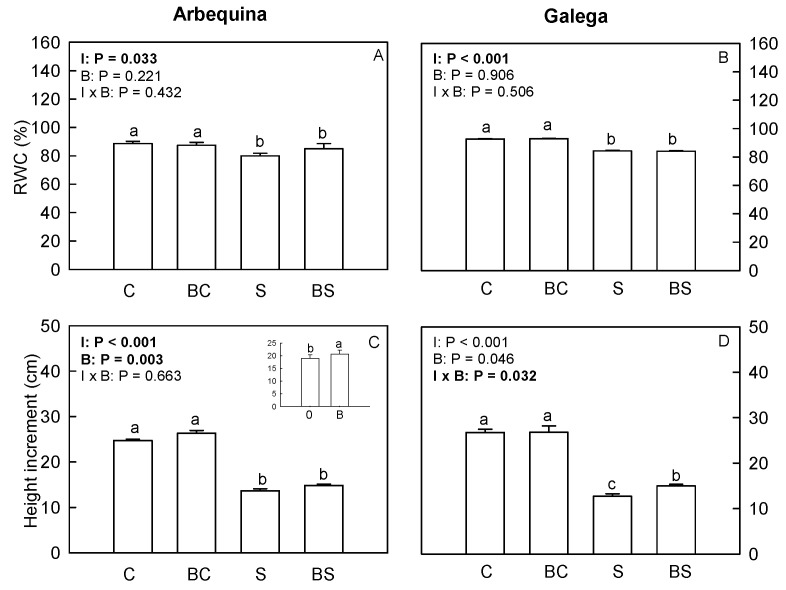
Leaf relative water content (RWC) (**A**,**B**) and plant height increment (**C**,**D**) in *O. europaea* plants of the treatments C (well-watered), BC (biostimulant + well-watered), S (water deficit), and BS (biostimulant + water deficit). Bars represent mean ± standard error (*n* = 5–10). The effect of the factor irrigation (I), factor biostimulant (B), and the interaction between the factor irrigation and biostimulant (I × B) are presented, and when the effect of each factor or the interaction is statistically significant (*p* ≤ 0.05), it appears in bold. Different letters indicate statistically significant differences (*p* ≤ 0.05). Significant differences among I × B refer to differences between C, BC, S, and BS treatments. Significant differences among the factor I refer to differences between 100% (treatments C and BC) and 50% irrigation (treatments S and BS). Significant differences among the factor B are shown in a chart at the top of the corresponding graph, and statistic letters refer to treatments without biostimulant (0: treatments C and S) and treatments with biostimulant (B: treatments BC and BS).

In both olive cultivars, plants exposed to water deficit (S and BS) presented an RWC lower (*p* ≤ 0.05) than the ones under well-watered conditions (C and BC) (Figure 1A,B).

Arbequina plants under well-watered conditions (C and BC) showed a height increment higher (*p* ≤ 0.05) than those under water deficit (S and BS) (Figure 1C). Additionally, Arbequina plants treated with biostimulant (BC and BS) showed a height increment higher (*p* ≤ 0.05) than those not treated with biostimulant (C and S) (Figure 1C).

In Galega, plants from the treatments C and BC showed the highest height increment, and those of the BS treatment presented a height increment higher than S treatment.

**Figure 2:**
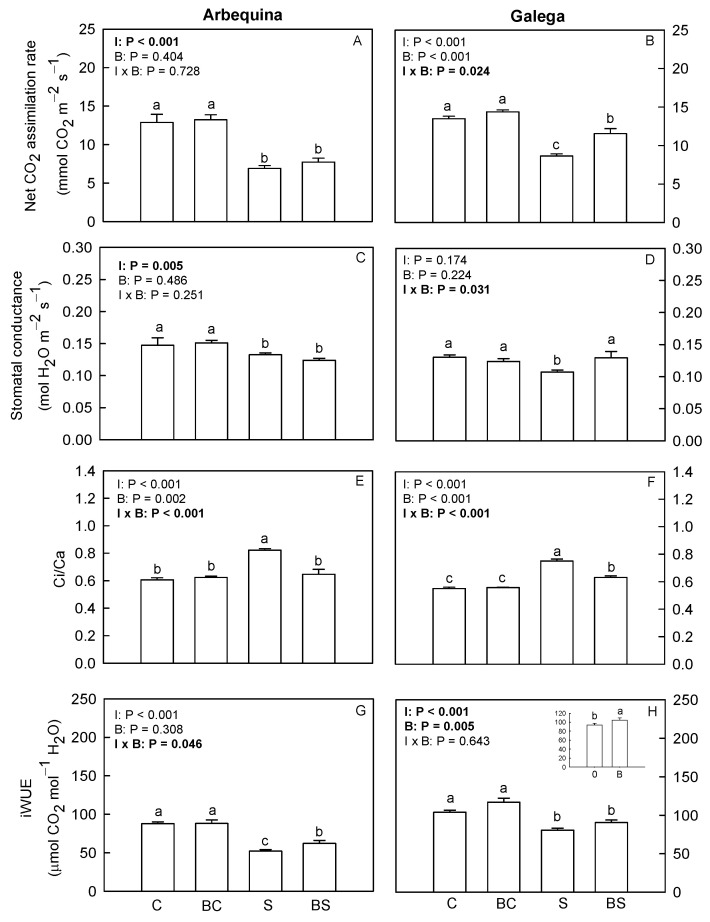
Net CO_2_-assimilation rate (**A**,**B**), stomatal conductance (**C**,**D**), ratio of intercellular CO_2_ and extracellular CO_2_ concentration (Ci/Ca) (**E**,**F**), and intrinsic water-use efficiency (**G**,**H**) in *O. europaea* plants of the treatments C (well-watered), BC (biostimulant + well-watered), S (water deficit), and BS (biostimulant + water deficit). Bars represent mean ± standard error (*n* = 6–9). The effect of the factor irrigation (I), factor biostimulant (B), and the interaction between the factor irrigation and biostimulant (I × B) are presented, and when the effect of each factor or the interaction is statistically significant (*p* ≤ 0.05), it appears in bold. Different letters indicate statistically significant differences (*p* ≤ 0.05). Significant differences among I × B refer to differences between C, BC, S, and BS treatments. Significant differences among the factor I refer to differences between 100% (treatments C and BC) and 50% irrigation (treatments S and BS). Significant differences among the factor B are shown in a chart at the top of the corresponding graph, and statistic letters refer to treatments without biostimulant (0: treatments C and S) and treatments with biostimulant (B: treatments BC and BS).

Figure 2 shows the gas-exchange parameters measured on olive leaves. In Arbequina, plants exposed to water deficit conditions (S and BS) showed a net CO_2_-assimilation rate and a stomatal conductance lower (*p* ≤ 0.05) than well-watered plants (C and BC). The water deficit treatment (S and BS) decreased (*p* ≤ 0.05) the transpiration rate in this cultivar. The application of biostimulant (BC and BS treatments) in Arbequina decreased (*p* ≤ 0.05) the transpiration rate. In Galega, plants from the C and BC treatments showed the highest values of net CO_2_-assimilation rate and transpiration rate. In turn, the plants of the BS treatment presented a net CO_2_-assimilation rate and transpiration rate higher than S plants. The highest stomatal conductance in Galega was found in plants from the treatments C, BC, and BS, while plants of the S treatment showed the lowest values. In Arbequina, plants exposed to the S treatment showed the highest Ci/Ca ratio. Plants from the treatments C, BC, and BS showed similar Ci/Ca values. For Galega, the plants of the S treatment also showed the highest Ci/Ca values, followed by the treatment BS. The lowest and a similar Ci/Ca ratio was found in plants of the C and BC treatments. Concerning the iWUE in Arbequina, plants of the C and BC treatments showed similar and the highest values. Plants of the BS treatment showed an iWUE higher than S. In Galega, biostimulant treatment (BC and BS) increased (*p* ≤ 0.05) the iWUE, but the application of the water deficit treatment (S and BS) reduced (*p* ≤ 0.05) this parameter.

**Figure 3:**
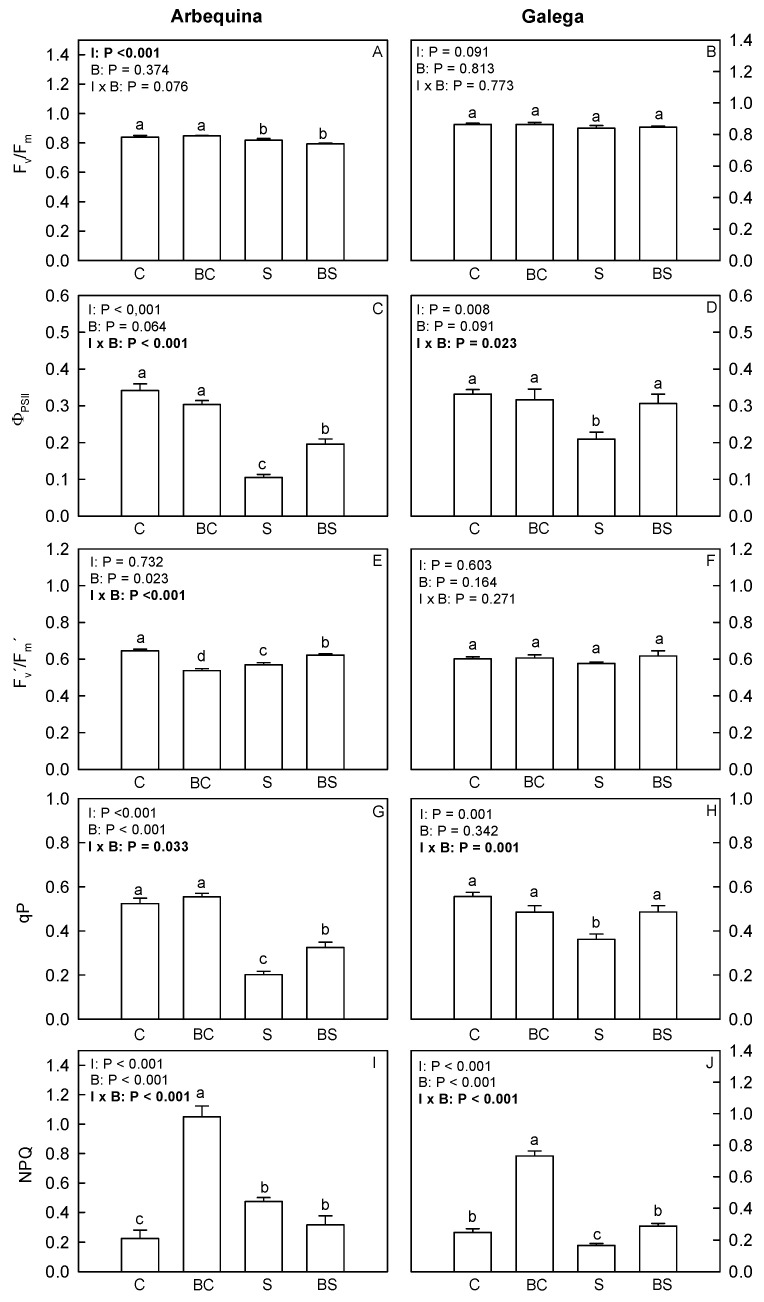
Maximum efficiency of PSII (F_v_/F_m_) (**A**,**B**), effective efficiency of PSII (Φ_PSII_) (**C**,**D**), efficiency of excitation energy capture by open PSII reaction centers (F_v_′/F_m_′) (**E**,**F**), photochemical quenching (qP) (**G**,**H**), and non-photochemical quenching (NPQ) (**I**,**J**) in *O. europaea* plants of the treatments C (well-watered), BC (biostimulant + well-watered), S (water deficit), and BS (biostimulant + water deficit). Bars represent mean ± standard error (*n* = 5–10). The effect of the factor irrigation (I), factor biostimulant (B), and the interaction between the factor irrigation and biostimulant (I × B) are presented, and when the effect of each factor or the interaction is statistically significant (*p* ≤ 0.05), it appears in bold. Different letters indicate statistically significant differences (*p* ≤ 0.05). Significant differences among I × B refer to differences between C, BC, S, and BS treatments. Significant differences among the factor I refer to differences between 100% (treatments C and BC) and 50% irrigation (treatments S and BS).

Concerning the chlorophyll *a* fluorescence parameters (Figure 3), the water deficit treatment (S and BS) reduces (*p* ≤ 0.05) the F_v_/F_m_ only in Arbequina plants. Also in Arbequina, plants from the treatments C and BC showed the highest Φ_PSII_ and qP. Plants from the BS treatment showed a Φ_PSII_ and qP higher than the S. In Galega, plants from the treatments C, BC, and BS presented the highest Φ_PSII_ and qP. In Arbequina, plants from the treatment C showed the highest F_v_′/F_m_′, and plants from the BC treatment showed the lowest F_v_′/F_m_′. In both cultivars, the highest NPQ values were found in plants of the BC treatment, while the lowest NPQ values were found in the C treatment in Arbequina and S treatment in Galega.

**Figure 4:**
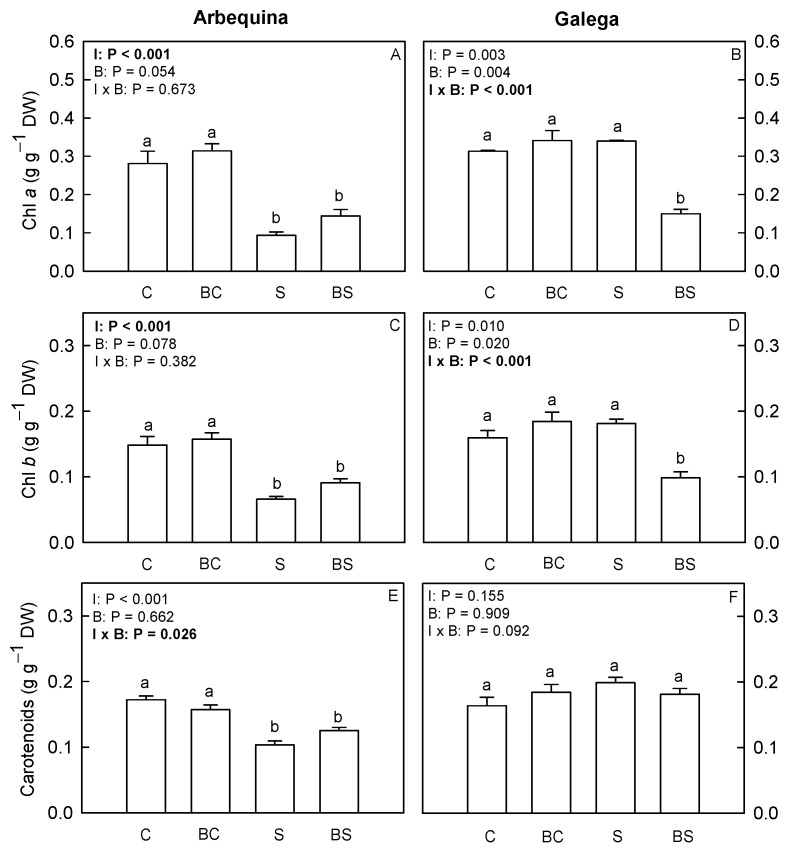
Chlorophyll *a* (**A**,**B**) and *b* (**C**,**D**), and carotenoid (**E**,**F**) contents in *O. europaea* plants of the treatments C (well-watered), BC (biostimulant + well-watered), S (water deficit), and BS (biostimulant + water deficit). Bars represent mean ± standard error (*n* = 6–8). The effect of the factor irrigation (I), factor biostimulant (B), and the interaction between the factor irrigation and biostimulant (I × B) are presented, and when the effect of each factor or the interaction is statistically significant (*p* ≤ 0.05), it appears in bold. Different letters indicate statistically significant difference (*p* ≤ 0.05). Significant differences among I × B refer to differences between C, BC, S, and BS treatments. Significant differences among the factor I refer to differences between 100% (treatments C and BC) and 50% irrigation (treatments S and BS).

Figure 4 presents the content of photosynthetic pigments in olive leaves. In Arbequina, the water deficit treatment (S and BS) reduced (*p* ≤ 0.05) the Chl *a* and Chl *b*. For Galega, plants of the BS treatment presented the lowest level of chlorophylls, while the plants under C, BC and S treatments showed the highest and similar levels of these pigments. Regarding carotenoids in Arbequina, the plants under S and BS treatments showed similar and the lowest levels of these pigments. The plants of the treatments C and BC presented similar and the highest levels of carotenoids.

### 2.2. Metabolite Profile

**Figure 5:**
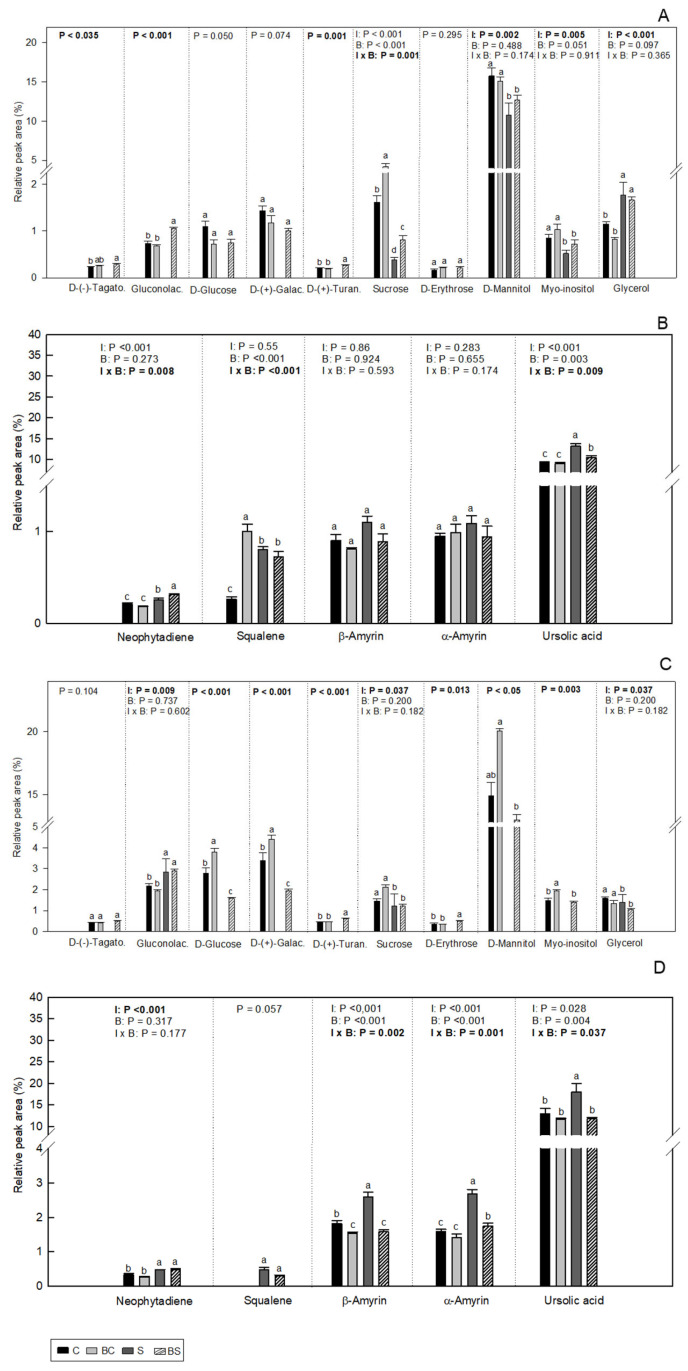
Carbohydrate (**A**,**C**) and terpene (**B**,**D**) relative abundance (%) in *O. europaea* plants of the cultivar Arbequina (**A**,**B**) and Galega (**C**,**D**) in the treatments C (well-watered), BC (biostimulant + well-watered), S (water deficit), and BS (biostimulant + water deficit). Bars represent mean ± standard error (*n* = 3–4). The effect of the factor irrigation (I), factor biostimulant (B), and the interaction between the factor irrigation and biostimulant (I × B) are presented, and when the effect of each factor or the interaction is statistically significant (*p* ≤ 0.05), it appears in bold. Different letters indicate statistically significant difference (*p* ≤ 0.05). Significant differences among I × B refer to differences between C, BC, S, and BS treatments. Significant differences among the factor I refer to differences between 100% (treatments C and BC) and 50% (treatments S and BS) irrigation. For the case of d-(−)-tagatofuranose, gluconolactone, d-glucose, d-(+)-galactose, d-(+)-turanose, and d-erythrose in Arbequina and d-(−)-tagatofuranose, d-glucose, d-(+)-galactose, d-(+)-turanose, d-erythrose, d-mannitol, and myo-inositol in Galega, one-way ANOVA was performed and significant differences (*p* ≤ 0.05) are marked in bold and indicated by different letters. d-(−)-Tagato.: d-(−)-Tagatofuranose; Gluconolac.: Gluconolactone; d-(+)-Galac.—d-(+)-Galactose; d-(+)-Turan.: d-(+)-Turanose.

Four classes of compounds were identified in olive plants of both cultivars, sugars, polyols, terpenes, and long-chain alkanes (Figure 5 and Table 1).

Sugars and polyols (d-(−)-tagatofuranose, gluconolactone, d-glucose, d-(+)-galactose, d-(+)-turanose, sucrose, d-erythrose, d-mannitol, myo-inositol and glycerol) were identified in olive leaves (Figure 5A,C). In Arbequina, the levels of d-(−)-tagatofuranose, gluconolactone, and d-(+)-turanose in plants of the BS treatment were significantly higher than in C plants. For sucrose, plants of the BC treatment showed the highest accumulation of this sugar. For the polyols, the water deficit treatment (S and BS) reduced (*p* ≤ 0.05) the levels of d-mannitol and myo-inositol, and increased (*p* ≤ 0.05) glycerol levels in Arbequina. In Galega, water deficit treatment (S and BS) increased (*p* ≤ 0.05) the levels of gluconolactone, while for sucrose and glycerol water deficit conditions (S and BS) induced a reduction (*p* ≤ 0.05) in these compounds. Plants of the treatment BC showed the highest levels of d-glucose, d-(+)-galactose, d-mannitol, and myo-inositol. In turn, Galega plants from the treatments BS showed the highest levels of d-(+)-turanose and d-erythrose.

In the leaves of both cultivars, five terpenes, neophytadiene, squalene, β- and α-amyrin, and ursolic acid, were identified (Figure 5B,D). In Arbequina, the plants of the BS treatment showed the highest levels of neophytadiene. Squalene was detected in the highest levels in plants of the BC treatments, while the S plants presented the highest levels in ursolic acid. In Galega, the plants of the S treatment showed the highest levels of β- and α-amyrin and ursolic acid. The water deficit treatment (S and BS) increased (*p* ≤ 0.05) the neophytadiene content.

Concerning the long-chain alkanes (C_30–50_), in Arbequina, plants of the S treatment showed the highest levels of alkanes 1, 3, 5, and 6. Biostimulant treatment (BC and BS) increases (*p* ≤ 0.05) the level of alkane 2, while water deficit treatment (S and BS) increased (*p* ≤ 0.05) the levels of alkane 4. In Galega, the plants of the treatments BC and S showed the highest levels of alkane 1, while the S plants showed the highest levels of alkane 5. Water deficit treatment (S and BS) increased (*p* ≤ 0.05) the levels of alkanes 3 and 6.

### 2.3. Relative Expression of OePIP1.1, OeDHN1, and OeHSP18.3

**Figure 6:**
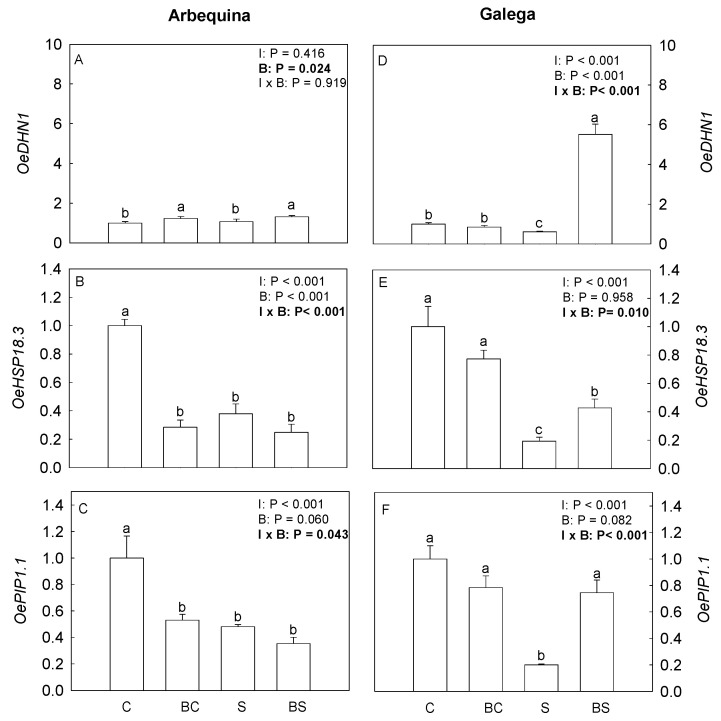
Relative expression of dehydrins *OeDHN1I* (**A**,**D**), small heat shock proteins *OeHSP18.3* (**B**,**E**), and aquaporins *OePIP1.1* (**C**,**F**) in *O. europaea* plants of the treatments C (well-watered), BC (biostimulant + well-watered), S (water deficit), and BS (biostimulant + water deficit). Bars represents mean ± standard error (*n* = 4–8). The effect of the factor irrigation (I), factor biostimulant (B), and the interaction between the factor irrigation and biostimulant (I × B) are presented, and when the effect of each factor or the interaction is statistically significant (*p* ≤ 0.05), it appears in bold. Different letters indicate statistically significant difference (*p* ≤ 0.05). Significant differences among I × B refer to differences between C, BC, S, and BS treatments. Significant differences among the factor B refer to treatments without biostimulant (treatments C and S) and treatments with biostimulant (treatments BC and BS).

Figure 6 shows the relative expression of *OeDHN1*, *OeHSP18.3,* and *OePIP1.1* genes. In Arbequina, biostimulant application (BC and BS) increased (*p* ≤ 0.05) the expression level of *OeDHN1*. The expression of *OeHSP13.3* and *OePIP1.1* was significantly higher in the plants of the C treatments, whilst the other treatments showed similar and lower values. In Galega, *OeHSP13.3* expression was significantly higher in plants of the treatments C and BC. The plants of the S treatment showed the lowest level of expression of this gene. For the *OePIP1.1* in Galega, the treatments C, BC, and BS showed an expression level significantly higher than the S treatment. In the same cultivar, the highest expression level of *OeDHN1* was found in plants of the BS treatments, while the lowest levels were in S plants.

### 2.4. Principal Component Analysis

**Figure 7:**
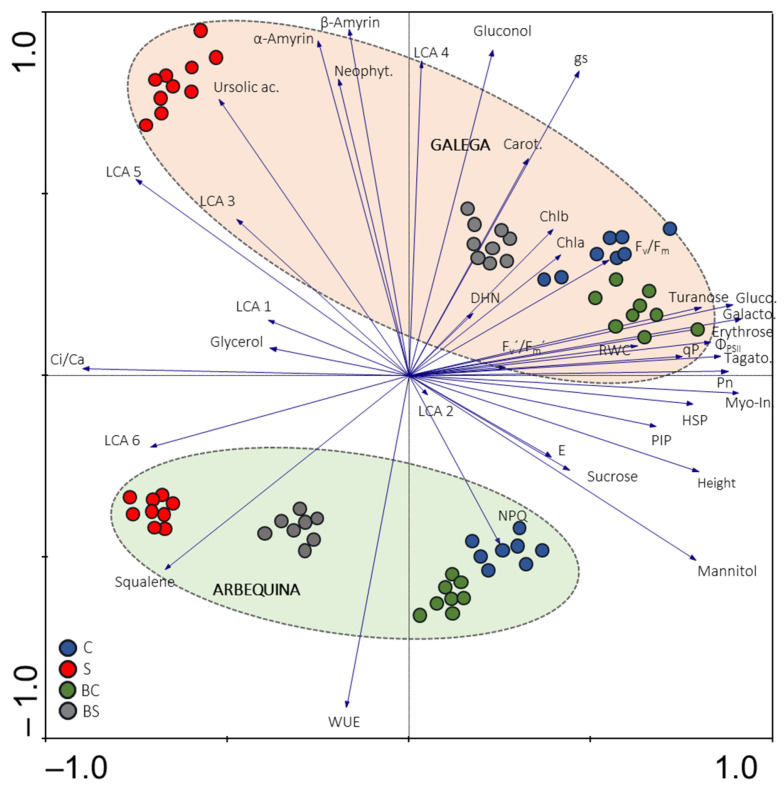
Principal component analysis plot (*x*-axis—first component PC1; and *y*-axis—second component PC2) of the physiological, molecular, and metabolomic data in olive leaves from both cultivars. PC1 explains 36% of the variance, while PC2 explain 23%. Circles with different colors depict sample scores of the different treatments. Ac.: acid; Carot.: carotenoids; DHN: *OeDHN1*; E: transpiration rate; Galacto.: d-galactose; Gluco.: d-Glucose; gs: stomatal conductance; Height: heigh increment; HSP: *OeHSP18.3*; LCA: long-chain alkane; Mio-In.: myo-inositol; Neophyt.: neophytadiene; PIP: *OePIP1.1*; P_n_: net CO_2_-assimilation rate; Tagato.: d-(−)- Tagatofuranose; WUE: intrinsic water-use efficiency.

To obtain an overview of olive physiological, metabolite, and gene expression profiles in response to biostimulant pre-treatment and irrigation level, a principal component analysis (PCA) was performed with both cultivars together (Figure 7). The multivariate analysis shows a clear separation between Galega and Arbequina cultivars and also treatments (C, BC, S and BS). Galega scores were positioned in the upper quadrant (I and II) far away from the ones of Arbequina localized in the lower quadrant (II and IV). Moreover, in Galega, scores of the C, BC, and BS treatments, besides being grouped separately, were closer and gathered in the upper right quadrant, while S scores were far away in the upper left quadrant. A different score distribution was found in Arbequina, being less spread in the lower quadrant. The BS and S scores were in the lower left quadrant with large separation, and the C and BC scores in the lower right quadrant with less separation between the two groups of scores.

## 3. Discussion

The use of more sustainable agricultural practices to improve plant growth and productivity is of high priority under the ongoing scenario of climate uncertainties [31]. Biostimulants have been described to improve yield and plant tolerance response to several stresses [15,16]. In the olive tree, studies conducted using biostimulants based on protein hydrolysates, *A. nodosum*, glycine betaine, kaolin, and *P. reactans* [17,18,19,20] showed positive effects on growth and yield. To our knowledge, this is the first report analyzing the effects of a commercial extract of *A. nodosum* (Fitoalgas Green^®^) on two olive cultivars under different irrigation levels. The pre-treatment of olive with *A. nodosum* extract favored some physiological processes under well-watered and water deficit conditions. Moreover, our results demonstrated that the effects of this biostimulant depend on cultivar and irrigation level. Arbequina and Galega cultivars exhibit a distinct response to biostimulant pre-treatment and irrigation (Figure 7).

### 3.1. Biostimulant Effects Under Optimal Irrigation Conditions

In olive, the pre-treatment with the biostimulant based on *A. nodosum* extract activated a diversity of processes that strengthen plant protection under optimal growth conditions (well-watered). This included the increase in thermal dissipation of excess excitation energy (NPQ), as well as the activation of DHN1 proteins (Arbequina cultivar) and antioxidant production, like myo-inositol and squalene (Arbequina cultivar). Moreover, this biostimulant also promoted plant height in Arbequina, and triggered the accumulation of carbohydrates (sucrose in Arbequina cultivar, and D-glucose, D-galactose, and mannitol in Galega cultivar), representing more energy available, for example, to maintain growth, or even a defense mechanism [32]. Another target of this biostimulant seems to be the photosynthetic process, acting in the upregulation of genes related to RuBisCO and carbonic anhydrase activity, promoting carbon fixation [33]. In olive, the stimulation of this key process was not so evident under well-watered conditions, but in the cultivar Galega the tendency to increase the net CO_2_ assimilation rate was reflected in improved iWUE. Overall, the biostimulant used in the present work seems to be efficient in providing some advantages that could contribute to a better development, and increased productivity under optimal growth conditions. However, several factors like species/cultivar, the mode of biostimulant application, and vegetative development status, can affect this response.

### 3.2. Biostimulant Effects under Water Deficit Conditions

Despite the high tolerance of olive trees to stress, drought conditions have been reported to reduce olive performance, decreasing productivity and changing oil quality [3]. Also, in the present work, the high temperatures (Supplementary Data, e.g., from July to the end of September 2020 the maximum T was above 41 °C, and the average temperature was above 25 °C) verified during all the period of water deficit negatively affected olive performance. However, the beneficial effect of biostimulant pre-treatment was found to counteract the negative effects of drought stress, promoting olive growth performance in a cultivar-dependent way (Figure 7).

A typical response of olive plants to drought conditions is the reduction in the stomatal aperture to prevent water loss [13]. In the present work, the moderate water stress imposed (50% field water content and stomatal conductance between 0.10 and 0.15 mol H_2_O m^−2^ s^−1^) [12,34] reduced the transpiration rate and stomatal conductance, and also the leaf relative water content (RWC) in plants of the S treatment. Higher reductions in water status accompanied with restrictions in the photosynthetic apparatus were reported for olive plants exposed to drought conditions [12,14]. For instance, Silva et al. [14] reported for the cultivar Cobrançosa a decrease from 90 to 66% of the leaf RWC after 30 days without irrigation (severe stress conditions), and Brito et al. [12] subjected olive trees of the cultivar Cobrançosa to three cycles of drought (withholding irrigation, severe stress) and verified a decrease in the RWC from 94 to 65%. Biostimulant pre-treatment (BS treatment) did not significantly change this response to moderate water stress but affected the stomatal aperture. For instance, in the cultivar Galega, the maintenance of a high stomatal aperture in plants of the BS treatment (similar to C and BC plants) could be related with the expression of *OePIP1.1*. In contrast, in plants of the S treatment *OePIP1.1* expression and gs were lower. These data suggest that pre-treatment with *A. nodosum* extract modulates *OePIP1.1* expression during water stress to regulate stomata opening in BS plants of the cultivar Galega. However, the contribution of this aquaporin to cellular water uptake and transcellular and intracellular transport [35] is not clear. In grapevine plants growing under field drought conditions, the treatment with a non-commercial *A. nodosum* extract improved the plant’s water potential [31]. However, in species like spinach, tomato, soybean, and hazelnut [16,36,37,38] alkaline *A. nodosum* extracts improved plant water status, acting in the modulation of ABA biosynthesis through the change in the expression of genes related to stomata aperture movements (*NCED3* and *MYB60*), resulting in stomata closure and water-loss prevention [16].

The photosynthetic reactions in olive trees were negatively affected by the water deficit treatment. However, the pre-treatment with *A. nodosum* improved the photosynthetic response and plant height increment more expressively in the cultivar Galega. Concerning the light-dependent reactions of photosynthesis, the F_v_/F_m_, an indicator of plant photosynthetic performance, was less sensitive to biostimulant and irrigation level, maintaining values above 0.76, which indicates no significant reduction in PSII efficiency, namely photoinhibition [39]. In turn, the effective efficiency of PSII (Φ_PSII_) dropped significantly due to water deficit stress, but *A. nodosum* pre-treatment’s positive effect was evident, since plants of the BS treatment presented a Φ_PSII_ higher than S plants. Moreover, in the cultivar Galega the pre-treatment with this biostimulant seems more efficient in helping to maintain a Φ_PSII_ similar to C and BC plants, possibly due to the improvement in open PSII reaction centers (qP). In the cultivar Arbequina, besides the qP, the efficiency of the excitation capture by open PSII reaction centers (F_v_′/F_m_′) was also favored by this biostimulant, contributing to sustaining a Φ_PSII_ in plants of the BS treatment higher than the S plants, but not at the level of the control plants (C and BC plants). Conversely, the reduction in the Φ_PSII_ of S plants might have compromised the availability of NADPH and ATP for the Calvin Cycle, contributing to the impairment of the net CO_2_-assimilation rate [6].

The chlorophyll *a* levels can also impact the Φ_PSII_ of olive plants [6]. In the cultivar Arbequina, the biostimulant did not affect chlorophylls, but the water deficit reduced these pigments, which possibly contributed to the drop in Φ_PSII_. The observed chlorophyll decrease in plants of the S and BS treatments could be a result of stress-induced degradation, possibly due to the reduction in carotenoids that play an important role in the protection against chlorophyll photodamage [40]. This can be supported by the increase in neophytadiene, a degradation product of chlorophyll [41] observed in plants of the S and BS treatments. In turn, the cultivar Galega appears more tolerant when only exposed to water deficit (plants of the S treatment), maintaining the chlorophyll at control levels, possibly due to carotenoid protection (via NPQ). Interestingly, in this cultivar, the biostimulant exerted a negative effect on chlorophyll content, but had no impact on the Φ_PSII_. All these data suggest that under water deficit stress this biostimulant acts on light-capture processes, through the increase in qP and F_v_′/F_m_′, to maintain the Φ_PSII_ and reduce chlorophylls pools, possibly by inducing a metabolic shift to the biosynthesis of other compounds more relevant to the stress response. In other species, like lettuce, endive, broccoli, celeriac, and spinach, the application of acid or alkaline commercial *A. nodosum* extracts increased not only the photosynthetic efficiency but also the level of chlorophylls [16,37,42], through the regulation of the dissipation of the excess of energy as heat (NPQ), upregulation of *Psb*, *VDE*, and *FIB1a* genes related to photoprotection mechanisms at PSII, downregulation of chlorophyll degradation genes (*AtCLH1* and *AtCLH2*), and increase in expression of betaine aldehyde dehydrogenase and choline monooxygenase genes [16,23,25].

Concerning the light-independent reactions of photosynthesis, the pre-treatment with *A. nodosum* was more effective in increasing net CO_2_ assimilation in the cultivar Galega, possibly due to a reduction in the biochemical restriction, allowing higher use of the intercellular CO_2_ (reduced C_i_/C_a_). These data suggest a putative action of this *A. nodosum* extract in the Calvin cycle enzyme activities, like RuBisCO. The work of Santaniello et al. [23] supports this hypothesis, demonstrating that acid extract of *A. nodosum* induces a protection mechanism that avoids a decline in the carboxylation capacity through the downregulation of RBCS1A and RCA, which codify respectively for a small subunit of RuBisCO and for a protein that catalyzes RuBisCO activation during photosynthesis. Additionally, *A. nodosum* was also effective in increasing the iWUE under water deficit conditions in both olive cultivars. In the cultivar Galega, iWUE improvement was possibly related to the increase in the net CO_2_ assimilation, but in the cultivar Arbequina it could be due to partial stomatal closure. These data are in line with previous studies on other species [23] and support that the use of *A. nodosum* extracts in agriculture can improve crop yields under the current depletion of water resource availability increasing drought tolerance [43].

Carbohydrates provide the energy for plant development and growth, and drought stress conditions usually induce an accumulation of reducing sugars (e.g., glucose and sucrose), and sugar alcohols (e.g., mannitol) that can act as osmoprotectants or as ROS scavengers [32]. In olive plants, sugar accumulation was reported in response to several abiotic stresses [44]. In contrast, in the present work the plants of the S treatment showed a reduction in the levels of main carbohydrates, possibly due to the reduction in the net CO_2_-assimilation rate, higher energy use to cope with the stress conditions, or metabolic shifts for the production of other stress protective compounds [45]. However, the pre-treatment with *A. nodosum* reverted this trend of response under water deficit condition, triggering the accumulation of some carbohydrates (such as d-(−)-tagatofuranose, gluconolactone, d-(+)-turanose and d-erythrose) in plants of the BS treatment. This effect of *A. nodosum* can result from the upregulation of genes related to carbohydrate biosynthesis and the downregulation of sucrose degradation genes [25]. Carbohydrate accumulation in olive plants during water deficit may help to maintain osmotic potential and cell turgor [46].

The stabilizing proteins DHN1 and HSP are involved in the olive plants’ response to drought, heat [6], and salinity [47]. In the present work, DHN1 was the most responsive to the pre-treatment with *A. nodosum* extract, particularly in the BS plants of the cultivar Galega, suggesting a more important role in stress response. In other species, Goñi et al. [37] and Vaseva et al. [48] demonstrated that *A. nodosum* extracts exert stress-mitigation effects that are linked with changes in the expression of dehydrin genes. The overexpression of the DHN1 protein in the BS plants of the cultivar Galega may provide more protection against cell damages caused by the drought stress, possibly through membrane stabilization by acting as chaperones preventing inactivation or aggregation of proteins under dehydration, by inhibiting ROS production at the source or by scavenging ROS [49]. Besides this protein, *A. nodosum* was also effective in maintaining an expression level of *OeHSP18.3* under water deficit conditions (BS plants) close to control plants (plants of the treatments C and BC) and above the plants of the treatment S also in the cultivar Galega. One mode of action of biostimulants (based on seaweeds) to enhance stress tolerance is inducing a higher number of HSPs, increasing the stability of proteins and membranes, leading to higher assimilate partitioning, water-use efficiency, and photosynthesis [50]. Altogether, these data indicated that this *A. nodosum* extract seems to exert control in the expression of these stress proteins, but at different intensities, contributing to protecting olive plants and promoting higher physiological performance under water deficit stress conditions. The higher effect of this *A. nodosum* extract on DHN1 expression and its relationship with stress protection deserves further studies.

To improve drought tolerance, some species such as olive tree invest in the strengthening of the cuticle structure by increasing the amount of several wax components [12,51,52]. In olive leaves, water deficit conditions alone (S treatment) increased the levels of some wax components, like long-chain alkanes, amyrins, and ursolic acid. Interestingly, the pre-treatment with *A. nodosum* extract, BC plants, seems not to favor this stress protection mechanism under water deficit conditions.

## 4. Material and Methods

### 4.1. Plant Material and Treatments

Three-year-old *Olea europaea* L. plants from the cultivars Arbequina and Galega were kindly provided by Viveiros Miguel Vaz (Semide, Coimbra, Portugal). Plants were transferred to black plastic pots of 5 L, with 22.5 cm diameter and 20 cm height, filled with turf (SIRO Turfa 30-0, Cantanhede, Portugal), and placed at the Botanical Garden of the University of Coimbra (Coimbra, Portugal). The temperature was monitored regularly with a portable USB data logger (LOG32TH, Dostmann, Wertheim, Germany) (Supplementary Data provides the monthly maximum, minimum, and average temperatures between April 2019 and September 2020). Olive plants were fertilized, and a source of boron (0.5% Borax, SPD, Porto, Portugal) was applied before flowering according to this culture’s agricultural practices. Since no significant phenological differences were observed between the cultivars, the treatments described below were applied at the same time in both cultivars. After one year of growth, plants of the two cultivars were randomly divided into two groups, one was sprayed with the biostimulant Fitoalgas Green^®^ (Tradecorp, Portugal, https://tradecorp.pt/product/fitoalgas-green/, accessed on 1 December 2023) and the other with distilled water. Fitoalgas Green^®^ is a 100% active, cold-processed extract of the seaweed *A. nodosum*, composed of 16.5% (*w*/*v*) dry matter and with a pH of 4.5. The beneficial effects of *A. nodosum* extracts on plants are thought to be triggered by a specific or by several bioactive compounds present in the extract, such as phenolic compounds (phlorotannins) and polysaccharides (like mannitol, alginic acid, fucoidan, ascophyllan, laminarin, and carrageenan) [16,22,53].

**Figure 8:**
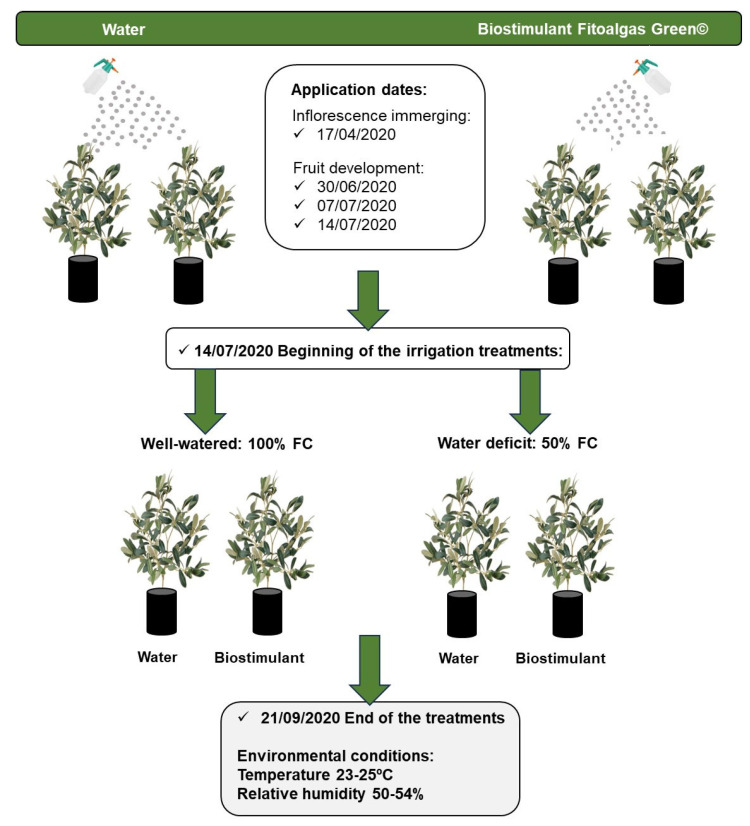
Schematization of the experiment. FC, field capacity.

The biostimulant was applied prior to flowering and three times during fruit development according to commercial instructions (Figure 8). Each plant was sprayed with 200 mL of 0.50 g/L extract. On the last day of biostimulant application (14 July 2020, Figure 7), all plants of the two groups, treated with biostimulant or water (control group), were placed at 100% field capacity (FC). The group of plants treated with biostimulant was divided into two subgroups: a) biostimulant + well-watered treatment (group BC—plants treated with biostimulant and watered at 100% FC, *n* = 8 plants for Arbequina cultivar and *n* = 9 plants for Galega cultivar), and b) biostimulant + water deficit treatment (group BS—plants treated with biostimulant and watered at 50% FC, *n* = 8 plants for Arbequina cultivar and *n* = 9 plants for Galega cultivar). The control group of plants sprayed with water was also divided into two subgroups: (a) without biostimulant + well-watered treatment (group C—plants not treated with biostimulant and watered at 100% FC, *n* = 8 plants for Arbequina cultivar and *n* = 8 plants for Galega cultivar), and (b) without biostimulant + water deficit treatment (group S—plants not treated with biostimulant and watered at 50% FC, *n* = 9 plants for Arbequina cultivar and *n* = 10 plants for Galega cultivar). After the day 14 July 2020, the water was replaced to 100 or 50% FC two times per week according to the treatment. Plants were maintained under these treatments for 69 days (Figure 8). Pots were randomly arranged and rotated weekly to minimize the effects of environmental heterogeneity.

In the last day of the experiment, photosynthesis was measured and leaves were collected to measure water status. Additionally, leaf samples were collected immediately, frozen in liquid nitrogen, and stored at −80 °C.

### 4.2. Plant Height, Water Status, Gas Exchange, Chlorophyll a Fluorescence, and Pigments

Plant height was measured before and after the experiment, and plant height increment was calculated.

Plant water status was determined by measuring the leaf relative water content (RWC) = (fresh weight − dry weight)/(turgid weight − dry weight) × 100. Fresh leaves were collected between 11 a.m. and 12 p.m., and the fresh weight was immediately determined. Turgid weight was determined after incubation of the leaves in water for 48 h at 4 °C in the dark. Dry weight was determined after drying the leaves at 80 °C for 10 days.

A portable photosynthesis system LI-6400XT (LI-COR, Lincoln, NE, USA) equipped with the leaf chamber 6400-40 LCF with a light source (LED-based fluorescence and light source accessory for the LI-6400) was used to measure the gas exchange and chlorophyll *a* fluorescence parameters. In situ leaf gas-exchange measurements (net CO_2_-assimilation rate (*Pn*), stomatal conductance (*g_s_*), transpiration rate (*E*), and the ratio of intercellular to extracellular CO_2_ concentration (C_i_/C_a_)) were taken between 9 a.m. and 12 p.m., under 400 μmol mol^−1^ of CO_2_, temperature of 23–25 °C, and light intensity of 800 µmol (photon) m^−2^ s^−1^. The iWUE was calculated as iWUE = *Pn*/*g_s_*. Chlorophyll *a* fluorescence was measured at the same time as gas exchange. In leaves adapted to growth light conditions, the steady-state fluorescence was averaged for 2.5 s, and after applying a saturating light pulse for 0.8 s the maximum fluorescence in light was determined. Leaves were then shaded and the F_0_′ was established. Then, leaves were adapted to dark (with leaf clips) for around 1 h and a saturating pulse of light (>5000 µmol photons m^−2^ s^−1^) was applied for 0.8 s. The maximum quantum efficiency of PSII (F_v_/F_m_ = (F_m_ − F_0_)/F_m_) was determined. The effective efficiency of PSII (Ф_PSII_ = (F_m_′ − F_s_)/F_m_′), efficiency of excitation energy capture by open PSII reaction centers (F_v_′/F_m_′ = (F_m_′ − F_0_)/F_m_′), photochemical quenching (qP = (F_m_′ − F_s_)/(F_m_′ − F_0_′)), and non-photochemical quenching (NPQ = (F_m_ − F_m_′)/F_m_′) were determined.

Photosynthetic pigments, chlorophyll *a* and *b*, and carotenoids, were determined in frozen leaves collected between 11 a.m. and 12 p.m. Leaf powder (~50 mg) was homogenized with 1 mL of acetone:50 mM Tris pH 7.8 mixture (80:20, *v*/*v*), and centrifuged at 4 °C, 10,000× *g* for 10 min. The supernatant was collected, and the absorbance read at 470, 537, 647, and 663 nm in a microplate reader EnSpire (PerkinElmer, Waltham, MA, USA), according to Sims and Gamon [54].

### 4.3. Metabolite Analysis

Lipophilic metabolites (sugars, polyols, terpenes, and long-chain alkanes) were extracted according to protocols already established for olive plants [55]. Leaves were collected between 11 a.m. and 12 p.m., immediately frozen in liquid nitrogen and stored at −80 °C. For the extraction of metabolites, three or four pools of frozen leaves (each pool consisted of 4 leaves from 2 plants) per treatment were made for the cultivars Arbequina and Galega, respectively. For each pool, leaves were ground, and 1 g was mixed with 20 mL of *n*-hexane. After 48 h at room temperature and magnetic stirring, the hexane was renewed, and the extraction continued for 24 h. The extract obtained in the two extractions was evaporated to dryness at low pressure with a rotatory evaporator. The dried extracts were then used for analysis. An aliquot of the leaf extract (200 μL of a solution of 10 mg mL^−1^) was mixed with 200 μL of tetracosane (internal standard, 0.45 mg mL^−1^), 250 μL of pyridine, 250 μL of *N*,*O*-bis(trimethylsilyl) trifluoroacetamide, and 50 μL trimethylsilyl chloride in a glass tube. After 50 min at 70 °C, the mixture was injected into the QP2010 Ultra Shimadzu (Shimadzu Corporation, Tokyo, Japan) equipped with a capillary J&W DB-5 column (30 m × 0.25 mm i.d. and a film thickness of 0.25 μm). The chromatographic conditions used are described in Dias et al. [55]. Briefly, samples were injected in a split ratio of 1:50, and helium was used as carrier (at 35 cm s^−1^). During the first 5 min, the initial temperature of the column was set to 80 °C, then the temperature was increased, first at 4 °C/min until 285 °C, followed by 2 °C/min until 300 °C, which was maintained for 5 min. The injector temperature was 250 °C, and the transfer-line temperature was 290 °C. The mass spectrometer worked in the electron impact (EI) mode with an energy of 70 eV and the data collected at a rate of one scan s^−1^ over a range of *m/z* between 33 and 750. The ion source was maintained at 250 °C. The chromatography lasted a total of 80 min.

The chromatographic peaks obtained in each chromatogram were identified using mass spectra databases (NIST14 Mass spectral and WILEY Registry^TM^ of Mass Spectra Data), or by comparison with the retention times and mass spectra of standard compounds analyzed using the same conditions described for the leaf extracts.

Leaf compound semi-quantification was performed by peak integration. For each chromatogram obtained, the area of each peak and the total peak area were calculated. The compound relative abundance (%) was then determined as follows: relative peak area average (%) = ((compound peak area/internal standard)/total peak area average) × 100]. The results were expressed as mean ± standard error of three or four independent analyses.

### 4.4. Protective Protein

To investigate the protective role of aquaporins, dehydrins, and small heat shock proteins, the corresponding gene expression was quantified. Leaves (6 to 8 leaves /plant) were collected between 11 a.m. and 12 p.m., immediately frozen in liquid nitrogen and stored at −80 °C. Leaf RNA was extracted according to Araújo et al. [6]. Briefly, 6–8 frozen leaves from each plant were ground and 100 mg of powder was mixed with 900 μL of extraction buffer (0.1 M Tris pH 8.0; 1.4 M NaCl; 20 mM Na_2_EDTA pH 8.0; 2% cetyltrimethylammonium bromide (*m*/*v*); 2% polyvinylpyrrolidone (*w*/*v*) and 0.3% β-mercaptoethanol) for 15 min at 65 °C with vortex mixing every 5 min, and then cooled on ice for 5 min. To the samples, 900 μL of chloroform:isoamyl alcohol (CIA, 24:1, *v*/*v*) was added followed by vigorous mixing, then samples were left on ice for 5 min and centrifuged at 16,000× *g* for 10 min at 4 °C. The upper phase was transferred to another tube, followed by adding 900 μL of CIA, and the mixing and centrifugation process was repeated twice. Then 0.1 volumes of 3 M sodium acetate and 2 volumes of absolute ethanol (−20 °C) were added to the samples followed by gentle mixing and the samples were incubated for 1 h at −20 °C. The samples were centrifuged at 16,000× *g* for 30 min at 4 °C and the supernatant was discarded. The pellet was washed with 400 μL of 70% ethanol (−20 °C), centrifuged at 16,000× *g* for 3 min at 4 °C and the supernatant was discarded. To the pellet, 250 μL of absolute ethanol was added, and centrifuged at 16,000× *g* for 3 min at 4 °C, the supernatant was discarded, and the pellet dried out. The pellet was re-suspended in 30 μL of Milli-Q water and stored at −80 °C. Samples were then treated with NZy DNase I (NZYTech™, Lisbon, Portugal) and cDNA was synthesized using the NZY First-Strand cDNA Synthesis kit (NZYTech™, Lisbon, Portugal) according to the manufacturer’s protocol. cDNA samples were diluted (1:20) in Milli-Q water and stored at −20 °C. All RT-qPCR reactions were performed using a CFX96 Touch Real-Time PCR (Bio-Rad Laboratories, Hercules, CA, USA). Each RT-qPCR reaction (10 μL) contained 5 μL of NZYSpeedy qPCR Green Master Mix (2×), ROX plus (NZYTech™, Portugal), 0.8 μL of F/R primer mix (10 μM), 2.5 μL of prediluted cDNA (1:20), and 1.7 μL of Milli-Q water. Amplification conditions were the following: 2 min at 95 °C followed by 45 cycles of 5 s at 95 °C and 20 s at 58 °C. The melting curve analysis ranged from 65 to 95 °C with incremental temperatures of 0.5 °C in 5 s/cycle. Real-time PCR Miner was used to calculate the efficiency of the primers and to determine cycle thresholds (CTs). Gene expression levels of *OePIP1.1*, *OeDHN1*, and *OeHSP18.3* were calculated relative to the control (C) and normalized with two housekeeping genes (*OeACT1* and *OeGAPB*, Araújo et al. [6]) according to the ΔΔCT method (Applied Biosystems, 2008), after CT transformation with efficiency values.

### 4.5. Data Analysis

The statistical analysis was performed with the SigmaStat program for Windows, version 4.0, integrated with SigmaPlot 11 (Systat Software, San Jose, CA, USA). A two-way ANOVA was performed to assess the effects of the irrigation, biostimulant, and interaction between these two factors. For the metabolite profile, one-way ANOVA was also performed when compounds were not detected in all the four treatments. When data were statistically different, the Holms Sidak Comparison Test was performed. When normality and equality of variances were not achieved, data were transformed. Whenever the assumptions were not verified, a non-parametric test (Kruskal–Wallis) was performed, followed by the post-hoc analysis (Dunn’s method). The significance level was 0.05. The multivariate analysis was carried out in Canoco for Windows Version 4.56 (Biometrics—Plant Research International, Wageningen, The Netherlands) [56].

## 5. Conclusions

The water deficit imposed negatively affected olive performance. However, the pre-treatment of olive plants with the *A. nodosum* extract Fitoalgas Green^®^ activated several molecular and physiological processes resulting in different response profiles that depend on the cultivar and irrigation level. In general, *A. nodosum* increased the olive physiological performance, leading to higher iWUE and growth. These benefits were more expressive under water deficit conditions, particularly in the cultivar Galega, which showed higher performance. Priming Galega plants with *A. nodosum* extract resulted in increased height increment, photosynthesis (light-dependent and -independent reactions) and iWUE under water deficit, through the maintenance of light-capture processes (Φ_PSII_ and qP), decrease of chlorophylls, and reduction of Calvin cycle restrictions. In Arbequina, *A. nodosum* extract effects were also found under water deficit conditions, acting positively at the growth level, iWUE, and promoting the light-dependent reactions of photosynthesis (Φ_PSII_, qP, and F_v_′/F_m_′). *A. nodosum* extract also seems to modulate the stomata opening, through the regulation of the expression level of *OePIP1.1* and maintenance of *OeDHN1* and *OeHSP18.3* genes upregulated, providing more stress protection, particularly in the cultivar Galega. Under well-watered conditions, *A. nodosum* extract effects were less pronounced, but also associated with inducing priming effects on protection/antioxidant mechanisms (NPQ and DHN1 in Arbequina cultivar, myo-inositol in Galega cultivar, and squalene in Arbequina cultivar), resulting in increased iWUE in the cultivar Galega and more energy availability (through carbohydrate accumulation) in both cultivars. All these improvements evidence the efficiency of this biostimulant, providing advantages that can contribute to better development and productivity, and conferring to primed olive plants a higher ability to overcome abiotic stress conditions, particularly during the establishment in the field. Further comparative studies might elucidate the evolution of the cultivars’ functional/metabolomic responses during longer-term periods of exposure with more sampling points. Also, the beneficial effects of this biostimulant on the olive fruit yield and oil quality remain a matter of debate. Nevertheless, the findings of this study contribute to the growing body of knowledge on sustainable agriculture and provide valuable information for farmers seeking to reduce their reliance on intensive irrigation methods.

## Figures and Tables

**Table 1:** Long-chain alkanes (LC Alk.) relative abundance (%) in *O. europaea* plants of the treatments C (well-watered), BC (biostimulant + well-watered), S (water deficit), and BS (biostimulant + water deficit). Values are mean ± standard error (*n* = 3–4). The effect of the factor irrigation (I), factor biostimulant (B), and the interaction between the factor irrigation and biostimulant (I × B) are presented, and when the effect of each factor or the interaction is statistically significant (*p* ≤ 0.05), it appears in bold. Different letters indicate statistically significant differences (*p* ≤ 0.05). Significant differences among I × B refer to differences between C, BC, S, and BS treatments. Significant differences among the factor I refer to differences between 100% (treatments C and BC) and 50% (treatments S and BS) irrigation. NS: not significant (*p* > 0.05).

Compound	Arbequina					Galega				
	C	BC	S	BS	*p*-Value	C	BC	S	BS	*p*-Value
LC Alk. 1	1.540 ± 0.037 ^c^	1.930 ± 0.054 ^b^	2.100 ± 0.182 ^a^	1.605 ± 0.097 ^b c^	**I × B: *p* = 0.002**	1.618 ± 0.070 ^b^	2.022 ± 0.107 ^a^	2.014 ± 0.066 ^a^	1.662 ± 0.036 ^b^	**I × B: *p* < 0.001**
LC Alk. 2	0.421 ± 0.029 ^a^	0.436 ± 0.013 ^a^	0.370 ± 0.040 ^b^	0.485 ± 0.022 ^b^	**I: *p =* 0.036**	0.346 ± 0.034 ^a^	0.433 ± 0.012 ^a^	0.419 ± 0.069 ^a^	0.485 ± 0.042 ^a^	NS
LC Alk. 3	3.016 ± 0.081 ^c^	3.761 ± 0.093 ^b^	4.541 ± 0.095 ^a^	3.089 ± 0.070 ^c^	**I × B: *p <* 0.001**	3.398 ± 0.077 ^b^	3.954 ± 0.354 ^b^	4.432 ± 0.150 ^a^	4.055 ± 0.219 ^a^	**I: *p =* 0.027**
LC Alk. 4	0.566 ± 0.019 ^a^	0.593 ± 0.044 ^a^	0.671 ± 0.049 ^a^	0.668 ± 0.055 ^a^	NS	0.957 ± 0.105 ^a^	0.786 ± 0.126 ^a^	0.947 ± 0.056 ^a^	0.816 ± 0.018 ^a^	NS
LC Alk. 5	5.636 ± 0.160 ^b^	5.985 ± 0.167 ^b^	7.672 ± 0.329 ^a^	5.667 ± 0.102 ^b^	**I × B: *p <* 0.001**	6.007 ± 0.419 ^b c^	5.291 ± 0.147 ^c^	9.584 ± 0.811 ^a^	6.520 ± 0.148 ^b^	**I × B: *p =* 0.014**
LC Alk. 6	0.402 ± 0.030 ^b^	0.438 ± 0.013 ^b^	0.622 ± 0.073 ^a^	0.430 ± 0.005 ^b^	**I × B: *p =* 0.032**	0.290 ± 0.069 ^b^	0.314 ± 0.021 ^b^	0.536 ± 0.103 ^a^	0.334 ± 0.014 ^a^	**I: *p =* 0.048**

## Data Availability

Data will be available as requested.

## Supplementary Materials

The following supporting information can be downloaded at: https://www.mdpi.com/article/10.3390/plants13202908/s1, Figure S1: Monthly maximum, minimum and average temperatures from 1 April 2019 to 30 September 2020. Red arrows indicated the beginning and end of the water deficit treatment (14 July 2020 to 21 September 2020).
