# Supplementary material for: Ascophyllum nodosum Extract Improves Olive Performance Under Water Deficit Through the Modulation of Molecular and Physiological Processes
Source: Plants (Basel). 2024 Oct 17;13(20):2908. doi: 10.3390/plants13202908 (PMC11511455; doi:10.3390/plants13202908)
Supplement: Supplementary file 1 [file plants-13-02908-s001.zip › plants-3106762-supplementary.pdf]

Supplementary Data

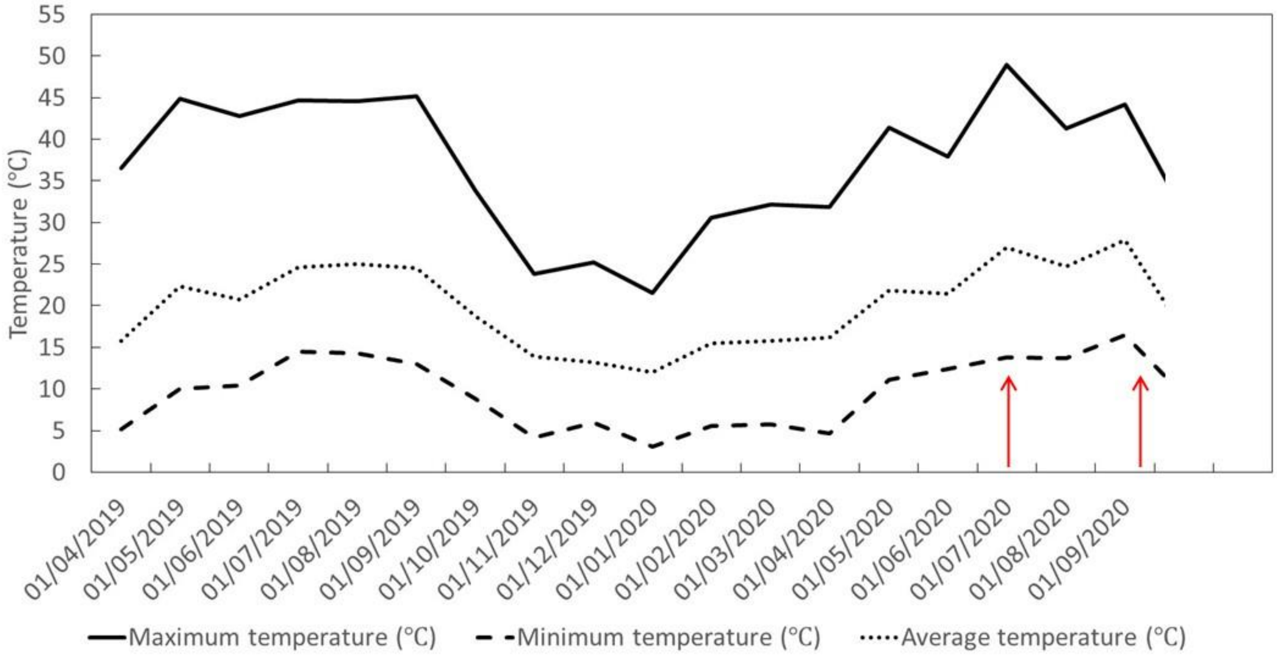

Figure S1 - Monthly maximum, minimum and average temperatures from 01/04/2019 to30/09/2020. Red arrows indicated the beginning and end of the water deficit treatment (14/07/2020 to 21/09/2020).
